# Executive Summary of the Guidelines on Stable Coronary Disease

**DOI:** 10.5935/abc.20150136

**Published:** 2015-10

**Authors:** Luiz Antonio Machado César, Antonio de Pádua Mansur, João Fernando Monteiro Ferreira

**Affiliations:** Instituto do Coração (InCor) HC-FMUSP, São Paulo, SP – Brasil

**Keywords:** Coronary Artery Disease, Diagnosis, Risk Factors, Physical Examination, Atherosclerosis

## Part I – Diagnosis and risk stratification

### Introduction

These guidelines aim to assist physicians, particularly cardiologists, to identify adults at high risk of coronary disease as early as possible, and to highlight its most common symptoms, especially coronary arery disease (CAD) symptoms.

According to Brazilian’s Unified Health System database (DATASUS), cardiovascular causes represent nearly 30% of all causes of death in Brazil^1^.

**Recommendation levels:**
- Class I: conditions for which there is conclusive evidence or general agreement that the procedure is useful/effective;
- Class II: conditions for which there is conflicting evidence and/or divergence of opinion about the usefulness/efficacy of the procedure;
- Class IIa: weight of evidence/opinion in favor of usefulness/efficacy. Approved by the majority of the professionals;
- Class IIb: safety and usefulness/efficacy is less well established, with no predominance of opinion in favor of the procedure;
- Class III: conditions for which there is evidence and/or general agreement that the procedure is not useful or effective and in some cases may be harmful;

**Evidence level:**
- Level A: data derived from multiple consistent, large randomized clinical trials and/or robust systematic meta‑analysis of randomized clinical trials.
- Level of evidence B: data derived from a less robust meta-analysis, a single randomized trial or nonrandomized (observational) studies.
- Level of evidence C: data derived from consensus opinion of experts.

### Diagnosis

#### Diagnosis of subclinical coronary artery disease

The risk of atherosclerotic disease may be measured by the sum of individual risks and by the synergism between the known risk factors for cardiovascular disease. Due to these complex interactions, an intuitive approach of risk attribution frequently lead to underestimation or overestimation of cases with higher or low risk, respectively.

##### Diagnosis of symptomatic patients

The approach proposed by Diamond and Forrester^2,3^ (Table 1): Level of recommendation I, evidence level B was considered for diagnosis.

**Table 1:** Pre-test probability of coronary artery disease in symptomatic patients by age and sex (Diamond/Forrester e CASS Data)

Age (years)	Nonanginal chest pain		Atypical angina		Typical angina	
	Male	Female	Male	Female	Male	Female
35	3-35	1-19	8-59	2-39	30-88	10-78
45	9-47	2-22	21-70	5-43	51-92	20-79
55	23-59	4-25	25-79	10-47	80-95	38-82
65	49-69	9-29	71-86	20-51	93-97	56-84

For the assessment of cardiovascular risk, the Brazilian Guidelines for Atherosclerosis Prevention and the V Brazilian Guidelines on Dyslipidemia and Atherosclerosis Prevention were used^4,5^. (Level of recommendation IIa, evidence level B).

#### Diagnosis of manifest coronary artery disease

##### History, physical examination, differential diagnosis

###### Definition of angina

Angina is a clinical syndrome characterized by pain or discomfort in any of the following regions: chest, epigastrium, mandible, shoulder, dorsum, or upper limbs. It is triggered or aggravated by physical activity or emotional stress and attenuated by nitroglycerin and its derivatives.

###### Clinical assessment of patients with chest pain

**a) Clinical history:** Detailed clinical history. Some characteristics should be carefully investigated to determine the probability of the presence of angina:

quality: constriction, tightness, heaviness, distress, suffocation, discomfort, burning, and stabbing; location: precordium, retrosternal area, shoulder, epigastrium, neck, hemithorax and dorsum; irradiation: upper limbs (right, left, or both), shoulder, mandible, neck, dorsum, and epigastrium; duration: seconds, minutes, hours, or days; triggering factors: exertion, sexual activity, position, eating habits, breathing, emotional component , and spontaneous; relieving factors: rest, sublingual nitrates, analgesic, food, antacids, position, and apnea; associated symptoms: sweating, nausea, vomiting, pallor, dyspnea, hemoptysis, cough, presyncope, and syncope.

An episode of angina lasts for a few minutes. It is generally triggered by exertion of emotional stress, and relieved by rest. The use of nitroglycerin, such as sublingual nitrate, relieves angina within approximately 1 min. Pain in the chondrosternal joints is rarely of cardiac origin.

The Canadian Cardiovascular Society (CCS) grading of angina pectoris^6^ is the most widely used classification of angina (Chart 1).

**Chart 1:** Canadian Cardiovascular Society grading of angina pectoris

Class I	Habitual physical activity, such as walking and climbing sairs, does not cause angina. Angina occurs during prolonged or strenuous physical activity.
Class II	Slight limitation for habitual activities. Angina during walking or climbing stairs rapidly, walking uphill, walking or climbing stairs after meals or in the cold, in the wind or under emotional stress, or within a few hours after waking up. Angina occurs after walking two blocks or climbing more than 1 flight of stairs in normal conditions.
Class III	Limitation of habitual activities. Angina occurs after walking one block or climbing 1 flight of stairs.
Class IV	Unable to carry on any habitual physical without discomfort. Angina symptoms may be present at rest.

**b) Physical examination:** Physical examination is usually normal in patients with stable angina. However, during an episode of angina, it may provide important evidence about the presence of absence of CAD. When physical examination is performed during an episode of pain, third heart sound (S3), fourth heart sound (S4) or gallop, mitral regurgitation, paradoxical splitting of the second heart sound (S2), and bibasilar crackles are suggestive and predictive indicators of DAC^7^. The occurrence of atherosclerosis in other regions, including decreased pulse in lower limbs, arterial hardening, and abdominal aneurysm, increase the likelihood of CAD.

###### Differential diagnosis of chest pain: associated conditions, and provoking and relieving factors of angina

In all patients, especially in those with typical angina, associated (simultaneous) diseases that can precipitate "functional" angina, i.e. myocardial ischemia in the absence of significant anatomic coronary obstruction, should be considered. These diseases generally cause myocardial ischemia either by increasing myocardial oxygen consumption or by decreasing the oxygen supply. An increase in oxygen consumption may be caused by hyperthermia, hyperthyroidism, and cocaine use. Obstructive sleep apnea should be seriously considered in patients with significant nocturnal symptoms.

##### Noninvasive tests

Additional tests in stable angina are based on the probability of CAD. After estimating the probability, it is categorized as low, intermediate, or high according to established values: 10%–90% in intermediate probability, < 10% in low probability, and > 90% in high probability cases.

Since overall mortality of patients with stable angina varies from 1.2% to 2.4% per year^8^, a diagnostic method that leads to a higher incidence of complications and death would be inappropriate.

###### Electrocardiogram

The test is indicated when a cardiac cause of chest pain is suspected (level of recommendation I, evidence level B).

###### Chest radiography

Chest radiography is indicated for patients with CAD and signs or symptoms of congestive heart failure (level of recommendation I, evidence level B), and patients with signs and symptoms of pulmonary disease (level of recommendation IIa, evidence level B).

###### Exercise treadmill test

The most predictive variables in the diagnosis of coronary obstruction are ST-segment depression ≥ 1 mm (measured at 0.80 seconds from the J-point), with a horizontal or descending pattern, and presence of anginal pain.

####### Exercise treadmill test for the diagnosis of coronary obstruction

**Level of recommendation I, evidence level B**
1. Intermediate probability
**Level of recommendation IIa, evidence level B**
1. Suspected vasospastic angina.
2. Coronary angiography for assessment of intermediate lesions.
3. Asymptomatic individuals with more than two risk factors.
**Level of recommendation IIb, evidence level B**
1. A high or low pretest probability of coronary obstruction, based on age, sex and symptoms.
2. Risk assessment for noncardiac surgery (in low cardiovascular risk).

**Level of recommendation III:** abnormalities: pre-excitation syndrome or Wolff-Parkinson-White syndrome, pacemaker rhythm, ST-segment depression >1 mm at rest, and complete left bundle-branch block.

######## Echocardiography

Echocardiography may help in the diagnosis^9^, by showing reversible and irreversible abnormalities in segmental motion in patients with clinical features of CAD.

**a) Stress echocardiography in chronic coronary atherosclerotic disease:** the test is used in diagnosis and prognosis, to assess the impact of revascularization therapies and myocardial viability, and to support therapeutic decisions. The test has good accuracy for induced myocardial ischemia in patients with intermediate or high pretest probability, with higher diagnostic sensitivity and specificity as compared with the exercise treadmill test^10^.

**b) Preoperative evaluation:** according to recommendations of the American College of Cardiology/American Heart Association (ACC/AHA) and the European Association of Cardiovascular Imaging (EACVI), dobutamine stress echocardiography has been valuable in preoperative risk stratification in patients with CAD^11^.

######## Radioisotopes

Aspects of myocardial perfusion, cellular integrity, myocardial metabolism, myocardial contractility, and global or segmental ventricular function are evaluated^12^. The radioisotope thallium-201 is less frequently used because of its association with higher radiation, and is indicated for the detection of ischemia concomitant with viable myocardium.

######## Coronary angiography

Coronary lesions are significant when one or more epicardial arteries are obstructed, with at least 70% stenosis and/or stenosis greater than 50% of the left main coronary artery. Assessment and measurement of obstructions are performed using coronary angiography (Chart 2).

**Chart 2:** Recommendations for coronary angiography in patients with coronary artery disease

Class I	Stable angina (CCS III or IV) despite clinical treatment (B)
	High risk in noninvasive tests, regardless of angina (B)
	Angina and cardiac arrest or severe ventricular arrhythmia survivors (B)
	Angina and symptoms/signs of congestive heart failure (C)
Class IIa	Patients with uncertain diagnosis after noninvasive tests, when the benefits of an accurate diagnosis outweigh the risks and costs of coronary angiography (C)
	Unable to undergo noninvasive tests due to physical disability, illness, or obesity (C)
	High-risk jobs that require an accurate diagnosis (C)
	Patients with uncertain prognostic information after noninvasive tests (C)
Class IIb	Multiple hospitalizations for chest pain, when a definitive diagnosis is considered necessary (C)
Class III	Significant comorbidities, when the risks of angiography outweigh the benefits of the procedure (C)
	Stable angina (CCS I or II) that responds to drug treatment and no evidence of ischemia in noninvasive tests (C)
	Preference to avoid revascularization (C)

CCS: Canadian Cardiovascular Society.

######## Cardiac computed tomography

There are two main modes of examinations using cardiac computed tomography that use different techniques and provide different information: the calcium score and coronary computed tomography angiography.

**a) Calcium score**

Quantification of coronary artery calcification using calcium score correlates with the atheroscleroctic load^13^.

**Level of recommendation I, evidence level A**

Asymptomatic individuals at intermediate risk using the overall risk score.

**Level of recommendation IIa, evidence level B**

Asymptomatic individuals at low risk using the overall risk score and family history of early CAD.

**Level of recommendation IIIa, evidence level B**
1. Asymptomatic patients at high risk of CAD or with known CAD.
2. Follow-up of coronary calcification progression.
3. Symptomatic patients.

**b) Coronary computed tomography angiography**

Coronary computed tomography angiography enables the noninvasive evaluation of the lumen of coronary arteries^14^.

The test is clinically indicated for symptomatic patients with conflicting results between ischemia and clinical tests.

**Level of recommendation IIa, evidence level A**

Suspected chronic CAD using:
a) Previous conflicting or inconclusive ischemia tests;
b) Continuous symptoms and ischemia tests with normal or inconclusive results.

**Level of recommendation IIa, evidence level B**

1. To assess the patency of grafts for myocardial revascularization in symptomatic individuals with pretest probability.

**Level of recommendation IIb, evidence level B**

1. Symptomatic individuals with intermediate probability of CAD and positive ischemia tests.
2. Symptomatic individuals with low probability of CAD and negative ischemia tests.
3. Assessment of in-stent restenosis in symptomatic individuals with intermediate pretest probability.

**Level of recommendation III, evidence level B**

1. Symptomatic individuals with high probability of CAD.
2. Initial evaluation of CAD in asymptomatic individuals, able to exercise and with interpretable electrocardiogram.
3. Follow-up of coronary atheroscleroctic lesions in asymptomatic individuals.

######## Cardiovascular magnetic resonance imaging

Magnetic resonance imaging is an excellent diagnostic method; it allows the assessment of cardiac and vascular anatomy, ventricular function, myocardial perfusion, and tissue characterization in an accurate, reproducible manner, in a single test^15^.

**a) Myocardial ischemia**

The protocols for the investigation of ischemia by magnetic resonance with myocardial perfusion are similar to those used in scintigraphy.

**b) Delayed enhancement**

The diagnosis and characterization of areas of myocardial infarction/necrosis/fibrosis using CMR is based on the delayed enhancement technique^16-18^.

c) Coronary magnetic resonance angiography

The clinical use of the test has been focused on the assessment of congenital anomalies and the origin and course of the coronary arteries^19^.

####### Recommendations for magnetic resonance imaging

######## Level of recommendation I, evidence level A

Evaluation of the global (left and right) ventricular function, volume, and mass

Detection of ischemia:

- Assessment of myocardial perfusion under stress using vasodilators.
- Assessment of ventricular contractility using dobutamine stress magnetic resonance.
- Detection and quantification of myocardial fibrosis and infarction.
- Assessment of myocardial viability.

######## Level of recommendation I, evidence level B

Differentiation between ischemic and nonischemic cardiopahty

- Coronary magnetic resonance angiography:
- Assessment of congenital anomalies.

#### Cardiovascular risk stratification in CAD

The strategies and methods used in the diagnosis of CAD also provide information on disease severity, with implications for complementary invasive methods, including coronary angiography, and therapeutic decision-making.

##### Exercise treadmill test for the prognosis of coronary atherosclerosis

**Level of recommendation I, evidence level B**

Patients with intermediate or high probability of CAD after initial evaluation; patients showing changes in symptoms.

**Level of recommendation IIb, evidence level B**

Patients with pre-excitation, ST-segment depression > 1 mm in echocardiogram at rest, pacemaker rhythm, and complete left bundle-branch block.

**Level of recommendation IIa, evidence level C**

Revascularized patients with symptoms suggestive of ischemia.

**Level of recommendation III, evidence level C**

Patients with severe comorbidities.

In patients with CAD who are able to reach stage 3 of the Bruce protocol, the annual mortality rate is approximately 1%, whereas in those unable to exceed 5 METs, the annual mortality rate is approximately 5%^20^.

Other high-risk variables include ST-segment depression in multiple leads, persistent ST-segment depression in recovery phase > 5 min, inadequate chronotropic response, fall in systolic blood pressure during physical exertion or a flat curve, and severe ventricular arrhythmia at low level of exercise in the presence of ST-segment depression or anginal pain.

###### Stress echocardiography

Echocardiography for CAD prognosis takes into account mainly the left ventricle function, and the presence or absence of myocardial ischemia induced by physical or pharmacological stress on echocardiography. In asymptomatic patients who have successfully undergone coronary artery bypass graft surgery (CABG), routine evaluation using stress echocardiography is not indicated. Other important variables for risk stratification include pulmonary uptake of thallium in myocardial perfusion scintigraphy, and the transient increase in the left ventricle.

#### Strategies for the diagnosis and stratification of coronary artery disease

The prognosis of CAD may also be based on the direct anatomical visualization of the coronary lesion by coronary angiography. Normal functional testing, performed with appropriate stress protocol yields the same prognosis as compared with the standard coronary angiography test.

## Part II – Drug Treatment

The main objectives of the treatment of CAD are to prevent myocardial infarction and decrease mortality, and to reduce symptoms and the incidence of myocardial ischemia, providing a better quality of life.

### Drug treatments to reduce the risk of myocardial infarction and mortality

#### Antiplatelet drugs

**a) Acetylsalicylic acid (ASA):** Level of recommendation I, evidence level A.

b) Thienopyridine derivatives:

**Clopidogrel:** Level of recommendation I, evidence level B. Indicated when aspirin is absolutely contraindicated, and associated with aspirin after stent implant for at least 30 days.

**Ticlopidine:** Level of recommendation IIa, evidence level B. Indicated when aspirin is absolutely contraindicated, and associated with aspirin after stent implant for at least 30 days.

**c) Dipyridamole:** Level of recommendation III, evidence level B.

**d) Anticoagulants:** should be used in combination with aspirin in**** case of high risk of thrombosis, especially after myocardial infarction. Level of recommendation I, evidence level A.

As an alternative to aspirin intolerance: Level of recommendation IIa, evidence level A.

For specific situations and after implantation of antiproliferative drugs-coated stent, follow the Brazilian Guidelines of Antiplatelet Agents and Anticoagulants in Cardiology.

#### Secondary prevention: Hypolipidemic agent

Lifestyle change (LC) is recommended for all patients with CAD (Chart 3).

**Chart 3:** Recommendations for drug therapy in dyslipidemias

Indications	Class-level of evidence
Statins are first choice treatment in primary and secondary prevention	I-A
Fibrate monotherapy or in combination with statins to prevent microvascular diseases in type 2 diabetes patients	I-A
Associations of ezetimibe or resins with statins when LDL-C target levels are not achieved	IIa-C
Association of niacin with statins	III-A
Omega-3 fatty acids for cardiovascular prevention	IIII-A

Source: Brazilian guidelines for cardiovascular disease prevention^10^.

#### Blockade of the renin–angiotensin system

**a) ACE inhibitors:** the benefits of ACE inhibitors in the treatment of CAD have been shown in clinical trials involving asymptomatic patients with reduced ejection fraction^21^ and patients with ventricular dysfunction after acute myocardial infarction^21,22^. They should be used routinely for ventricular dysfunction, and/or heart failure, and/or diabetes mellitus management^23,24^. Level of recommendation I, evidence level A.

It should be used routinely in all patients with CAD: Level of recommendation IIa, evidence level A.

**b) Angiotensin receptor blockers:** alternative therapy for patients intolerant to ACE inhibitors, since no study has been conducted on the use of this group of drugs in stable coronary disease. In other situations, angiotensin receptor blockers have provided no additional benefits over those of ACE inhibitors, which can decrease the incidence of infarction.

### Treatment to reduce symptoms and myocardial ischemia

**a) Beta-blockers:** beta-blockers are drugs of choice, to be administered alone or in combination with other antianginal drugs. Indicated as first-line agents in patients with stable angina without previous myocardial infarction and/or left ventricle dysfunction^25^. Level of recommendation I, evidence level B.

- First-line agents in patients with stable angina within 2 years of myocardial infarction and/or left ventricle. Level of recommendation III, evidence level C.
- For symptomatic relief in patients with vasospastic angina: Level of recommendation III, evidence level C.

**b) Calcium-channel blockers:** heterogeneous group of drugs with pharmacological effects that include smooth muscle relaxation, afterload reduction, and negative inotropic effects (some formulations). On the other hand, they are contraindicated in case of ventricular dysfunction (verapamil and diltiazem)^26^.

– First-line agents for symptomatic relief in patients with vasospastic angina. Level of recommendation IIa, evidence level B.
– In symptomatic patients with stable angina on beta-blockers (dihydropyridines). Level of recommendation I, evidence level B.
– In symptomatic patients with stable angina on beta-blockers (verapamil or diltiazem). Level of recommendation III, evidence level B.
– In patients with stable angina and contraindications to beta-blockers (preferably verapamil or diltiazem). Level of recommendation I, evidence level B.
– In symptomatic patients with stable angina (fast-acting ihydropyridines). Level of recommendation III, evidence level B.

**c) Nitrates:**

– **Fast-acting nitrates:** for symptomatic relief of acute angina. Level of recommendation I, evidence level B.
– **Long-acting nitrates:** continuous use of long-acting nitrates leads to drug tolerance.
– First-line agents in patients with stable angina. Level of recommendation III, evidence level C.
– Third-line agents in stable angina patients who still have symptoms even after using other antianginal agents associated. Level of recommendation IIa, evidence level B.
– For symptomatic relief in patients with vasospastic angina after using calcium-channel blockers. Level of recommendation IIa, evidence level B.

**d) Trimetazidine:** drug with metabolic and anti-ischemic effects and no effect on cardiovascular hemodynamics^27^.

– In symptomatic patients with stable angina on beta-blockers alone or in combination with other antianginal agents. Level of recommendation IIa, evidence level B.
– In patients with stable angina and left ventricle dysfunction associated with optimized medical therapy. Level of recommendation IIa, evidence level B.
– In patients with stable angina during myocardial revascularization procedures (percutaneous or surgical). Level of recommendation IIa, evidence level B.

**e) Ivabradine:** a specific sinus node I_*f*_ current i inhibitor, which specifically decreases the heart rate^28^.

– In symptomatic patients with stable angina on beta-blockers alone or with other antianginal agents, and heart rate > 70 bpm. Level of recommendation IIa, evidence level B.
– In symptomatic patients with stable angina who are intolerant to beta-blockers alone or with other antianginal agents. Level of recommendation IIb, evidence level B.
– In patients with stable angina, left ventricle dysfunction (LVEF < 40%) and heart rate ≥ 70 bpm under optimized medical therapy. Level of recommendation IIa, evidence level B.

**f) Ranolazine:** piperazine derivative. Similar to trimetazidine, it protects patients from ischemia by increasing glucose metabolism and decreasing fatty acids oxidation. However, its major effect appears to be the inhibition of late sodium current^29^.

**Figure 1:**
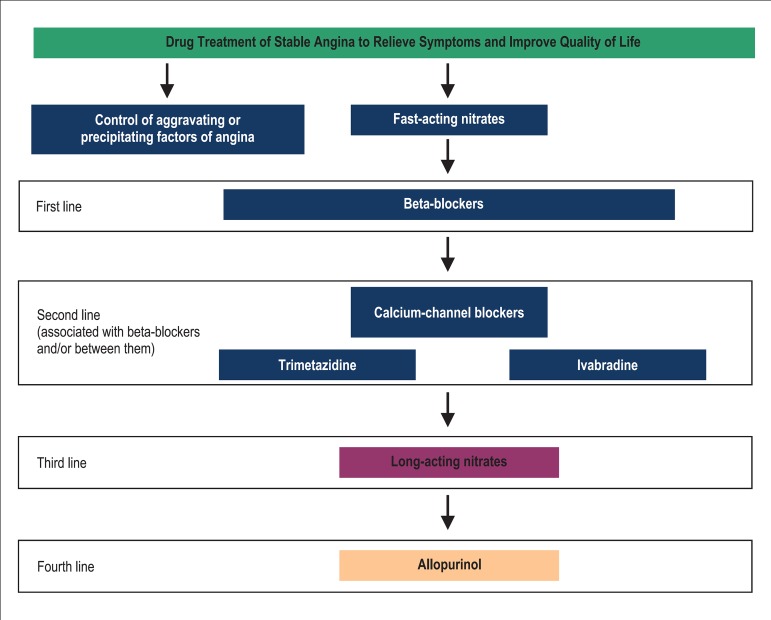
Algorithm for drug treatment of stable angina with antianginal drugs to relieve symptoms and improve quality of life. Details, levels of recommendation and evidence level: see the corresponding text.

**Figure 2:**
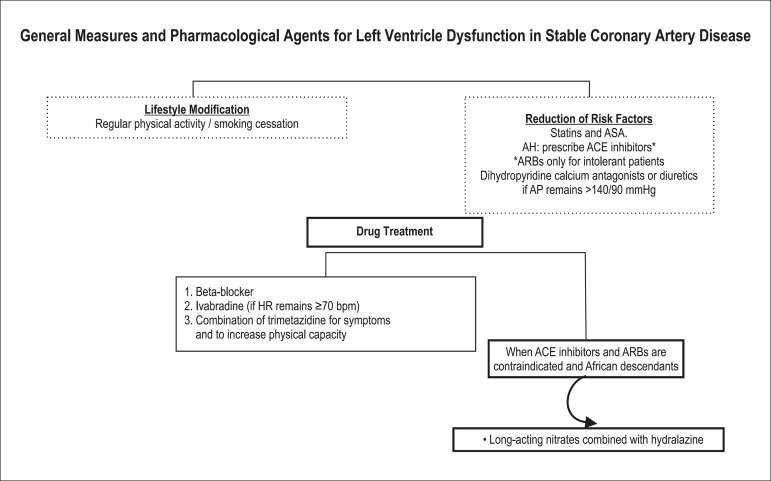
Algorithm for reduction of cardiovascular events in the presence of left ventricular dysfunction. Details, levels of recommendation and evidence level: see the corresponding text. ASA: Acetylsalicylic acid; AH: Arterial hypertension; ACE inhibitors: Angiotensin-converting enzyme inhibitors; ARB: Angiotensin receptor blocker I; AP: Arterial pressure; HR: Heart rate.

Figures 1 and 2 depict algorithms that facilitate understanding of drug therapy options in stable CAD.

## Part III – Treatment with invasive measures

### Treatment with invasive measures

#### Direct surgical revascularization

The Guidelines on Myocardial Revascularization^30^ cover the procedure techniques, alternatives, and current practices. They also briefly review classic studies, comparing surgical treatment strategies with clinical treatment and percutaneous coronary intervention.

##### Main indications for direct revascularization

**Level of recommendation I**

Left main coronary artery stenosis ≥ 50% or equivalent conditions (left descending anterior and circumflex arteries in the ostium, or before the exit of important branches). Evidence level A.

Proximal stenosis (> 70%) in the three main arteries with or without involvement of proximal left anterior descending artery, especially in patients with ejection fraction < 50% or functional evidence of moderate to severe ischemia. Evidence level B.

Stenosis in two main vessels, with proximal left anterior descending artery lesion in patients with ejection fraction < 50% or functional evidence of moderate to severe ischemia. Evidence level B.

**Level of recommendation IIa**

Left internal mammary artery graft in patients with significant stenosis (> 70%) in proximal left anterior descending artery and evidence of extensive ischemia, aiming to improve survival. Evidence level B.

Coronary artery by-pass surgery instead of percutaneous coronary intervention in patients with multivessel CAD and diabetes mellitus, particularly in those who underwent internal mammary artery grafting with revascularization to the left anterior descending artery. Evidence level B.

**Level of recommendation III**

Asymptomatic patients with normal ventricular function, without extensive areas of ischemia or involvement of the left anterior descending artery. Evidence level C.

Asymptomatic patients without significant anatomical lesions (< 70%, or < 50% of the left main coronary artery) or functional lesions (e.g., fractional flow reserve > 0.8 or mild ischemia in noninvasive tests). Evidence level C.

Involvement of one or two arteries, except for the proximal left anterior descending artery, with no evidence of relevant ischemia in functional tests, and presence of perfusion in small areas of viable myocardium. Evidence level B.

Moderate lesions (between 50% and 60%) except in left main coronary artery, without moderate ischemia in functional tests.

Insignificant lesions (< 50%).

##### The "Heart Team" concept for myocardial revascularization

###### Class I

A team made up of clinical cardiologists, cardiac surgeons and interventional cardiologists is recommended to individualize the indication for the treatment of left main coronary artery lesions or complex CAD. Evidence level C^31^.

#### Catheter-based revascularization: clinical indications

##### Revascularization vs. drug treatment (Figure 3)

**Figure 3:**
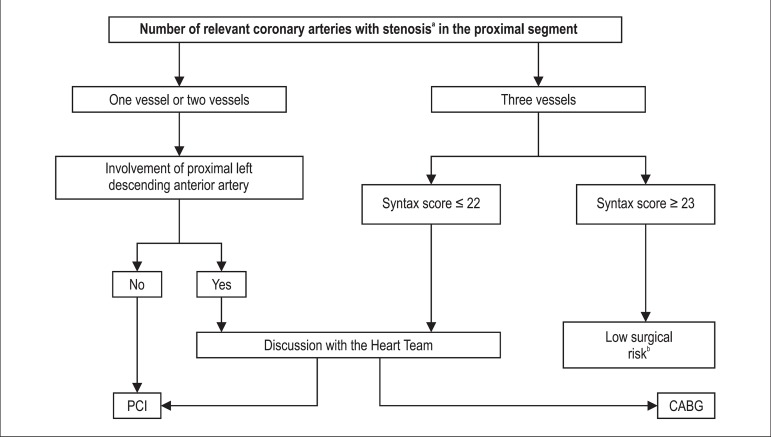
Percutaneous coronary intervention (PCI) or coronary-artery bypass grafting (CABG) in stable coronary atheroscleroctic disease without involvement of left main coronary artery. a≥ 50% stenosis and confirmation of ischemia, lesion > 90% confirmed by two physicians or fractional flow reserve of 0.80; bCABG is the preferred option in most patients, unless in case of comorbidities or other particularities that require discussion with the Heart Team. Adapted from: 2010 Guidelines on myocardial revascularization of the European Society of Cardiology and the European Association for Cardio-Thoracic Surgery.

###### Percutaneous coronary intervention vs. clinical treatment

To date, no study has demonstrated that percutaneous coronary intervention in patients with CAD improves survival rates^32^.

###### Appropriate use of revascularization

####### Patients with three-vessel disease

The coronary artery bypass surgery is the preferred strategy for three-vessel disease patients with increased age, low ejection fraction, renal dysfunction, peripheral vascular disease, diabetes mellitus, or Syntax score > 22.

### Special situations

#### Patients with diabetes mellitus

Diabetes mellitus is an increasingly prevalent condition associated with increased risk of cardiovascular complications, especially late mortality.

##### Indications for myocardial revascularization

###### Comparison of revascularization strategies in diabetic patients with multi-vessel CAD

Sensitivity analysis showed that the superiority of coronary artery bypass surgery was more evident in individuals with high Syntax score (> 33), with no significant difference between the low score and intermediate score groups^33^.

###### Aspects of percutaneous coronary intervention in diabetes mellitus patients

Drug-eluting stents are recommended to reduce restenosis and the need of a new target vessel revascularization^34,35^.

The dual antiplatelet therapy with aspirin and a P2Y12 receptor blocker is an essential component of drug regiments for perioperative and postoperative periods. Patients who receive drug-eluting stents should use the therapy for 12 months, and those who receive non-drug-eluting stents should use it for 1 month.

##### Patients with previous revascularization

The main indications for revascularization are persistence of symptoms, despite optimized medical therapy and/or prognosis.
